
JOURNAL INFORMATION
==============================
NLM Title Abbreviation: Behav Neurol
Journal ID: Behav Neurol
NLM Journal ID: 8914585
Journal ID: BN

Behavioural Neurology

ISSN: 0953-4180
EISSN: 1875-8584
Publisher: Wiley

ARTICLE INFORMATION
==============================
PMCID: PMC5434408
PMID: 21422556
DOI: 10.3233/BEN-2010-0296
Article ID: 476127
Article version: 1
Subjects: Research Article

Simple Spans in Deaf Signers and Hearing Non-Signers

Andin Josefine *
Rönnberg Jerker
Rudner Mary
Linnaeus Centre HEAD Swedish Institute for Disability Research Department of Behavioural Sciences and Learning Linköping University Linköping Sweden
*Josefine Andin: josefine.andin@liu.se
Print publication date: 2010
Electronic publication date: 2011 Mar 7
Volume: 23
Issue: 4
First page: 207
Last page: 208
Received 2011 Jan 31; Accepted 2011 Jan 31
Copyright: Copyright © 2010 Hindawi Publishing Corporation and the authors.
Copyright year: 2010
License: This is an open access article distributed under the Creative Commons Attribution License, which permits unrestricted use, distribution, and reproduction in any medium, provided the original work is properly cited.
License URL: https://creativecommons.org/licenses/by/3.0/

==============================
Behavioural Neurology 23 (2010) 207–208
DOI 10.3233/BEN-2010-0296
IOS Press

### Simple spans in deaf signers and hearing non-signers

Josefine Andin*, Jerker Rönnberg and Mary Rudner
*Linnaeus Centre HEAD, Swedish Institute for Disability Research, Department of Behavioural Sciences and Learning, Linköping University, Linköping, Sweden*

Short-term memory is traditionally assessed by digit span tests. Deaf signers (DS) have repeatedly been reported to perform at lower levels on this test than hearing non-signers (NH [3]), despite equal performance on more complex working memory tasks and other cognitive tasks. Hearing signers have also been shown to perform at lower levels when they are tested on sign language compared to spoken language, suggesting that the differences between DS and HN are dependent on language rather than on deafness per se [4,7].

Sign languages are the natural mode of communication for many deaf persons and are fully fledged languages that can be described using the same terminology as spoken language [8]. That means that sign languages have phonology, morphology, syntax and prosody as well as their own grammar and vocabulary. However, there are some differences between spoken and signed languages that might influence short-term memory.

Four commonly proposed explanations for these differences are (1) articulation rate, (2) phonological similarity effects, (3) differences in sensory memory traces and (4) temporal order effects: It has been suggested that short-term memory is restricted to the amount of words that can be articulated within two seconds [2]. Given that individual signs take longer to *articulate* than individual spoken words, this might affect rehearsal rate. According to the *phonological similarity effect*, items that are phonologically similar encode similar traces in the phonological loop, giving rise to confusable traces, that compromise short-term memory performance (for review see [2]). In most sign languages digits are considered to be phonologically similar while digits in most spoken languages are dissimilar, rendering shorter spans for signed languages. *Sensory memory traces* from auditory information have longer durability than from visual information [6,10]. Thus, memory traces from spoken (auditory) stimuli can be more effectively used than memory traces from signed (visual) stimuli. It has been argued that participants perform at their best when they are tested in their preferred language modality, which would be using spoken stimuli and oral recall for non-signers and signed stimuli and recall for signers. However, when persons using different modalities are to be compared we argue that it is more important to use modality neutral stimuli and recall. Finally, short-term memory tasks generally require remembering a sequence of words in a specific order. As the auditory system is better at retaining temporal order than the visual system [9], this also introduces a difference between signers and speakers in terms of recall. When free recall instead of serial recall is used differences between groups disappear [3].

Some of the differences presented above can be associated with differences in neural correlates. Generally, the same fronto-parietal networks are activated for both deaf signers and hearing non-signers during working memory tasks, but with some sign specific differences. Using fMRI Bavelier et al. [3] showed that the same areas were activated during serial recall in both deaf signers and hearing non-signers, but there was a difference in the reliance of functional components during the course of the task. During the encoding phase of

*Corresponding author. E-mail: josefine.andin@liu.se.

ISSN 0953-4180/10/$27.50 © 2010 – IOS Press and the authors. All rights reserved the task the two groups showed similar recruitment of inferior frontal areas, but during the later stages of rehearsal the hearing non-signers had a significantly higher overall activation and during recall the signers had a higher overall activation. They concluded that signers rely on passive memory storage to a higher extent than the non signers. Further, Pa et al. [5] have reported net activation in auditory cortex for non-signers, probably related to auditory processing. For the signers net activation was found in superior parietal lobes and in the temporo-occipital regions (*cf.* [7]). It was suggested that this might reflect a higher reliance on spatial orientation for order information in signers, which in turn would reflect the higher degree of spatial components in sign language compared to spoken language.

In an ongoing study we investigated span size in DS and HN, while keeping presentation and recall regimes constant, by the use of visual presentation and typed recall [1]. We found significant group differences on digit span, which we believe can be attributed to the phonological similarity effect for digits, but no differences on letter span. We argue that there are two explanations for the lack of differences on letter span: 1) The phonological similarity for letters is lower than for digits in sign language, and 2) the presentation and recall regimes reduce differences in sensory memory stores and reduces the ability of DS to take advantage of longer lasting auditory memory traces.

We suggest that when comparing short term memory in deaf signers and hearing non-signers modality neutral span tests should be used in order to make the conditions as equal as possible. This can be done by designing the material so that 1) the to-be-remembered items are presented at the same rate for all participants; 2) items that are phonologically dissimilar are used in both languages (e.g. letters instead of digits); 3) material is visually presented and typed recall is used; 4) free recall rather than serial recall is used.

### References

[1] J. Andin, J. Rönnberg and M. Rudner, *Language Modality Specific Effects on Simple Spans in Deaf Signers and Hearing Non-signers*, Paper presented at the Second Meeting of the Federation of the European Societies of Neuropsychology, Amsterdam, The Netherlands, 22–24 September 2010.

[2] A. Baddeley, Working memory and language: an overview, *J Commun Disord* **36**(3) (2003), 189–208.

[3] D. Bavelier, E.L. Newport, M. Hall, T. Supalla and M. Boutla, Ordered short-term memory differs in signers and speakers: implications for models of short-term memory, *Cognition* **107**(2) (2008), 433–459.

[4] M. Boutla, T. Supalla, E.L. Newport and D. Bavelier, Short-term memory span: insights from sign language, *Nat Neurosci* **7**(9) (2004), 997–1002.

[5] J. Pa, S.M. Wilson, H. Pickell, U. Bellugi and G. Hickok, Neural organization of linguistic short-term memory is sensory modality-dependent: evidence from signed and spoken language, *J Cogn Neurosci* **20**(12) (2008), 2198–2210.

[6] J. Ronnberg and K. Ohlsson, Channel capacity and processing of modality specific information, *Acta Psychol* (*Amst*) **44**(3) (1980), 253–267.

[7] J. Ronnberg, M. Rudner and M. Ingvar, Neural correlates of working memory for sign language, *Brain Res Cogn Brain Res* **20**(2) (2004), 165–182.

[8] M. Rudner, J. Andin and J. Ronnberg, Working memory, deafness and sign language, *Scand J Psychol* **50**(5) (2009), 495–505.

[9] M.M. Smyth, D.C. Hay, G.J. Hitch and N.J. Horton, Serial position memory in the visual-spatial domain: reconstructing sequences of unfamiliar faces, *Q J Exp Psychol A* **58**(5) (2005), 909–930.

[10] G. Sperling, The information available in brief visual presentations, *Cognitive Psychology* **74** (1960), 1–29.
